# Model-Based Analyses for the Causal Relationship Between Post-stroke Impairments and Functional Brain Connectivity Regarding the Effects of Kinesthetic Illusion Therapy Combined With Conventional Exercise

**DOI:** 10.3389/fnsys.2021.804263

**Published:** 2022-01-10

**Authors:** Yu Miyawaki, Masaki Yoneta, Megumi Okawada, Michiyuki Kawakami, Meigen Liu, Fuminari Kaneko

**Affiliations:** ^1^Department of Rehabilitation Medicine, Keio University School of Medicine, Tokyo, Japan; ^2^Neurorehabilitation Research Center, Kio University, Nara, Japan; ^3^Research Fellow of Japan Society for the Promotion of Science, Tokyo, Japan

**Keywords:** clinical trial, functional connectivity, kinesthetic illusion therapy, spasticity, stroke

## Abstract

**Aims:** Therapy with kinesthetic illusion of segmental body part induced by visual stimulation (KINVIS) may allow the treatment of severe upper limb motor deficits in post-stroke patients. Herein, we investigated: (1) whether the effects of KINVIS therapy with therapeutic exercise (TherEx) on motor functions were induced through improved spasticity, (2) the relationship between resting-state functional connectivity (rs-FC) and motor functions before therapy, and (3) the baseline characteristics of rs-FC in patients with the possibility of improving their motor functions.

**Methods:** Using data from a previous clinical trial, three path analyses in structural equation modeling were performed: (1) a mediation model in which the indirect effects of the KINVIS therapy with TherEx on motor functions through spasticity were drawn, (2) a multiple regression model with pre-test data in which spurious correlations between rs-FC and motor functions were controlled, and (3) a multiple regression model with motor function score improvements between pre- and post-test in which the pre-test rs-FC associated with motor function improvements was explored.

**Results:** The mediation model illustrated that although KINVIS therapy with TherEx did not directly improve motor function, it improved spasticity, which led to ameliorated motor functions. The multiple regression model with pre-test data suggested that rs-FC of bilateral parietal regions is associated with finger motor functions, and that rs-FC of unaffected parietal and premotor areas is involved in shoulder/elbow motor functions. Moreover, the multiple regression model with motor function score improvements suggested that the weaker the rs-FC of bilateral parietal regions or that of the supramarginal gyrus in an affected hemisphere and the cerebellar vermis, the greater the improvement in finger motor function.

**Conclusion:** The effects of KINVIS therapy with TherEx on upper limb motor function may be mediated by spasticity. The rs-FC, especially that of bilateral parietal regions, might reflect potentials to improve post-stroke impairments in using KINVIS therapy with TherEx.

## Introduction

Spasticity is frequently represented as the main symptom of motor deficits accompanied by paralysis, especially in the chronic phase in patients after stroke. The disorganization of afferent signals from peripheries or compensational signals from the brain to the peripheries is thought to cause spasticity. Motor deficits of the upper limb can impair patients’ daily lives after stroke, and solving this problem is important in rehabilitation medicine. We have encountered an event in which spasticity masks potential motor functions as if the cause of spasticity exists in a different system from motor paralysis. In fact, some patients’ motor accuracies improve immediately after the target muscle’s spasm is suppressed by therapy for spasticity, such as reinforced stretching, for a muscle that can interfere with accurate movement.

Spasticity disturbs the skilled and gross motor performance of the upper limbs (Pundik et al., 2019). For these motor deficits, studies have proposed some effective therapies, such as constraint-induced movement therapy (Wolf et al., 2006), mental practice (Page et al., 2007), or robot therapy (Klamroth-Marganska et al., 2014), which can have moderate effects (Pollock et al., 2014). However, these therapies require patients to perform voluntary movements or motor imagery, and severe cases with strong difficulties in such performances are often excluded. Therefore, an effective therapy for severe cases is required, for which a novel therapy utilizing kinesthetic illusion of segmental body part induced by visual stimulation (KINVIS) was proposed (Kaneko et al., 2016a, 2019; Aoyama et al., 2020; Okawada et al., 2020).

KINVIS is defined as the psychological phenomenon in which a resting person feels as if his/her own body part is moving or feels the desire to move a body part while watching a movie of that body part moving (Kaneko et al., 2007). We previously demonstrated that primary motor cortex excitability is enhanced during/after KINVIS (Kaneko et al., 2007, 2016b; Aoyama et al., 2012), as shown in the motor imagery of hand or finger movements without performing a voluntary movement (Kaneko et al., 2003, 2014; Yahagi et al., 1996). Moreover, previous studies revealed that motor-related areas, such as the premotor, superior, or inferior parietal cortex, were activated when experiencing KINVIS more than observing a similar movement (Kaneko et al., 2015; Shibata and Kaneko, 2019). These psychological experiences and neurological effects may contribute to recovering post-stroke motor deficits. For example, mirror therapy (Altschuler et al., 1999) that can induce kinesthetic illusions such as KINVIS has a moderate effect on motor deficits (Dohle et al., 2009; Pollock et al., 2014). In terms of interhemispheric inhibition (Murase et al., 2004; Nowak et al., 2009), it is noteworthy that KINVIS requires no voluntary movements even in a non-paralyzed upper limb. Since the KINVIS paradigm enables a person to passively experience finger or hand movements as a kinesthetic illusion, it has the potential to work as a treatment for severe cases.

Based on these findings, the KINVIS therapy was proposed and a clinical trial was conducted for 10 days (Kaneko et al., 2019). In this trial, 11 patients with a severe paretic upper limb in the chronic phase underwent KINVIS therapy and conventional therapeutic exercise (TherEx). The KINVIS therapy comprised a combination of KINVIS and neuromuscular electrical stimulation (NMES). Participants observed prerecorded finger flexion and extension movements of a virtual upper limb displayed on a monitor parallel to and above their own actual limbs. When participants observed the movements, electrical stimulations on the finger extensor muscles were combined to provide proprioception matched with the visual movements. This therapy was undertaken for 20 min, after which TherEx was performed for 60 min. Before and after this 10-day intervention, upper limb motor functions, spasticity, and resting-state brain functional connectivity (rs-FC) were assessed (i.e., pre- and post-tests). This clinical trial produced two main findings.

First, a previous study (Kaneko et al., 2019) had demonstrated significant improvements in the Fugl-Meyer Assessment (FMA), Action Research Arm Test (ARAT), and modified Ashworth Scale (MAS) scores after the interventions (see Supplementary Table 1). Interestingly, the improvement of the MAS score reached a minimum of clinically important differences (i.e., reduction of one or more; Barros Galvão et al., 2014). Given that this trial included only patients in the chronic phase, the natural recovery after stroke may not sufficiently explain these improvements. Although this clinical trial was undertaken only for 10 days, other therapies, such as mirror or robot therapy, require longer-term interventions (e.g., >6 weeks; Page et al., 2007; Dohle et al., 2009; Klamroth-Marganska et al., 2014). According to these facts, the effects of the KINVIS therapy with TherEx might have been observed by bringing out the masked potential functions of paretic upper limbs rather than directly recovering their motor deficits. Patients with severe motor deficits in the chronic phase often have severe spasticity, which can disturb the motor performance. In this trial, all patients had a MAS score of ≥2 (i.e., severe spasticity; Kaneko et al., 2019). Considering that significant improvements in the MAS scores were observed in this trial, the improvements in motor functions might have been induced through improvements in spasticity.

As the other finding, the previous clinical trial demonstrated significant correlations between rs-FC and FMA/ARAT scores (Kaneko et al., 2019). These motor function scores were correlated with the rs-FC between the inferior parietal sulcus in the affected (aIPS) and unaffected (uIPS) hemispheres, between the supramarginal gyrus in the affected hemisphere (aSMG) and the cerebellar vermis, and between the inferior parietal lobule in the unaffected hemisphere (uIPL) and dorsal premotor cortex in the unaffected hemisphere (uPMd). Although the causal relationships of these correlations remain unclear, these rs-FC can help capture characteristics in severe cases who participated in the previous trial (Kaneko et al., 2019). However, the previous study analyzed these correlations independently but did not consider spurious correlations. Because some regions composed of these rs-FC were similar to each other (i.e., parietal regions), the influences of their spurious correlations should be investigated. This investigation may contribute to exploring the characteristics of patients with the possibility of improved motor functions using KINVIS therapy with TherEx.

Taken together, although the KINVIS therapy may allow us to treat severe cases, further analyses of the data from the previous clinical trial (Kaneko et al., 2019) can help unravel the effects of its therapy. These analyses may lead to a hypothesis that should be examined in large-scale future clinical trials. Before proceeding to an ensuing trial, therefore, we sought to address the unresolved issues by performing path analyses in structural equation modeling with data from the previous clinical trial (Kaneko et al., 2019), i.e., reuse of data. First, we conducted a path analysis comprising the mediation model to investigate whether the improvements in upper limb motor functions after KINVIS therapy with TherEx were induced through improvements in spasticity. Second, we performed a path analysis comprising the multiple regression model to investigate the relationship among the three rs-FC (i.e., aIPS-uIPS, aSMG-Vermis, and uIPL-uPMd) and motor functions before intervention and without spurious correlations among them. Finally, we conducted a path analysis comprising the multiple regression model to investigate the baseline characteristics of rs-FC in patients with the possibility of improving their motor functions. This study is expected to help develop a novel treatment for patients with severe motor deficits.

## Methods

### Previous Clinical Trial

#### Participants

We utilized the data from a previous clinical trial (Kaneko et al., 2019) of KINVIS therapy with TherEx, because we aimed to investigate improvements in motor functions and correlations with rs-FC observed in this trial, with 11 patients registered (seven men and four women; five right hemiparesis and six left hemiparesis; mean age = 54.7 years, SD = 10.8). The patients had a unilateral stroke without cortical involvement, the ability to flex the paretic fingers voluntarily without extension, time from stroke onset of >4 months, and the ability to walk independently with or without assistance. Moreover, the patients had not received other special rehabilitations or treatments for paretic upper limbs, such as transcranial magnetic stimulation, repetitive facilitative exercise, or botulinum toxin injection within 3 months. In summary, the patients had severe hemiparesis of the upper limbs in the chronic phase.

#### Interventions

**Figure 1:**
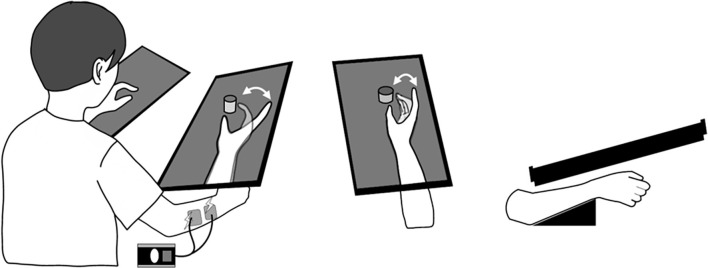
KINVIS therapy setup. When participants observed the movie of prerecorded finger flexion and extension movements, the electrical stimulation on the finger extensor muscles was combined to provide proprioception matched with visual finger movements. The virtual upper limb on the monitor was set parallel to and above an actual limb.

The patients participated in the clinical trial, with assessments undertaken before and after this 10-day intervention (pre- and post-tests), comprising KINVIS therapy and conventional TherEx. In KINVIS, patients underwent a combination therapy of KINVIS and neuromuscular electrical stimulation (Figure 1). Electrical stimulation was combined to provide proprioception matched with visual finger movements and was applied at an intensity higher than the finger extensor muscles’ motor threshold (motor threshold = 1.0–1.2 times higher; frequency = 20 Hz; pulse width = 50 μs) when watching a finger-extension phase movie. In one intervention (1 day), patients underwent KINVIS therapy for 20 min and TherEx for 60 min post KINVIS therapy.

#### MRI Data Acquisition and Preprocessing

All magnetic resonance imaging data (MRI) were obtained using a 1.5-T MRI scanner with a head coil (Optima 450w, GE Healthcare, Chicago, IL, USA). Resting-state functional MRI (fMRI) was performed to measure rs-FC. For this scanning session, a gradient-echo echo-planar sequence was used (3.75 × 3.75 × 3.5 voxels; echo time = 40 ms; repetition time = 2,500 ms, flip angle = 85), in order to collect BOLD contrast data of 244 slices. Moreover, a T1-weighted MRI scan was acquired. T1-weighted structural images were acquired as an anatomical reference (0.4688 × 0.4688 × 1.4 mm voxels; echo time = 3.076 ms; repetition time = 8.368 ms; flip angle = 12). MRI data acquisition was performed before and after the intervention.

All data preprocessing were carried out using CONN toolbox (Whitfield-Gabrieli and Nieto-Castanon, 2012) implemented on MATLAB (Mathworks Inc., Natick, MA, USA). CONN is a conjunction in Statistical Parametric Mapping 12 (SPM12; Well-come Department of Imaging Neuroscience, London, UK); therefore, the following preprocessing pipeline is SPM12 compliant. Image preprocessing consisted of the following: (1) reducing the signal for the first four slices for magnetization equilibrium effects and adaptation of the subjects to the circumstances; (2) flipping of all structural/functional data of right-hand affected patients using non-rigid reflection along the x-axis, in order to align the affected hemisphere on the left side; (3) functional realignment and unwarping; (4) slice-timing correction applied to the images; (5) creating binary masks in gray matter, white matter, and cerebrospinal fluid images using structural segmentation; (6) denoising using outlier detection thresholded 97th percentile in normative samples of framewise displacement; (7) normalization to the EPI image template conforming to the Montreal Neurological Institute (MNI) space (2 mm iso voxels); (8) spatial smoothing with an isotropic Gaussian kernel of 7-mm full width at half maximum; and (9) band-pass filtering with a setting of 0.01–0.08 Hz.

After the data preprocessing, regions of interest, which were defined as spherical seed regions with a radius of 6 mm, were created in the MNI space on the affected and unaffected hemispheres (also see Kaneko et al., 2019 for more detailed methods).

### Outcome Measures

In the present study, we adopted the data of the FMA, ARAT, MAS, and rs-FC (aIPS-uIPS, aSMG-Vermis, and uIPL-uPMd) for analyses using the FMA and ARAT scores as the main outcomes for upper limb motor function. The MAS score was analyzed to investigate the influence of spasticity and consisted of a total score of the 2nd to 5th fingers and wrist flexor muscles. The MAS is an ordinal scale with scores of 0, 1, 1+, 2, 3, and 4. For analyses using the MAS score, scores of 1+, 2, 3, and 4 were transformed to 2, 3, 4, and 5, respectively (transformed scores: 0, 1, 2, 3, 4, and 5). As the outcomes of brain activities, we focused on z-scores of the aIPS-uIPS, aSMG-Vermis, and uIPL-uPMd rs-FC (see “Introduction” section). These z-scores were calculated for each of the pre- and post-tests.

### Statistics

Since all participants had the same FMA scores in the wrist (0 for all) and hand (1 for all) subscales, variances in the FMA scores reflected the shoulder, elbow, and forearm gross motor functions (scores of the shoulder/elbow/forearm subscales). We analyzed three variables using the ARAT scores: total, finger-related (ARAT-F), and shoulder-related (ARAT-S) scores. The ARAT-F refers to the total score of the grasp, grip, and pinch subscales, mainly reflecting the hand and finger skilled motor functions. The ARAT-S score refers to the gross movement subscale score, mainly reflecting the upper limb gross motor functions (especially the shoulder). Using these variables, we performed three main investigations. First, conducting a path analysis comprising the mediation model, we investigated the relationship between the effects of the KINVIS therapy with TherEx on upper limb motor function and spasticity. Second, performing a path analysis comprising the multiple regression model, we investigated the relationship among the three rs-FC (i.e., aIPS-uIPS, aSMG-Vermis, and uIPL-uPMd) and motor function prior to intervention and without spurious correlations among them. Finally, conducting path analysis comprising the multiple regression model, we investigated baseline characteristics of rs-FC in patients who can potentially improve their motor functions.

#### Correlation Analyses and t-Tests

**Figure 2:**
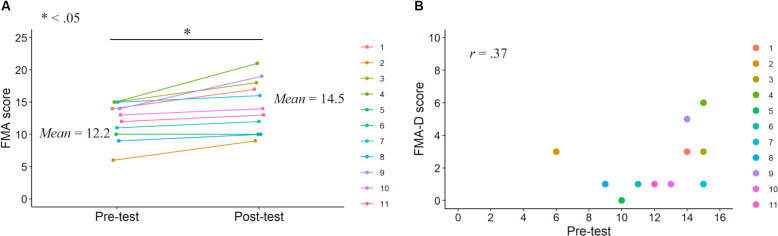
FMA scores in the pre- and post-tests. FMA score improvements after the KINVIS therapy with TherEx reported by Kaneko et al. (2019) and their individual differences **(A)**. The relationship between the FMA-D (degree of the improvements from the pre- to post-tests) and pre-test scores **(B)**.

**Figure 3:**
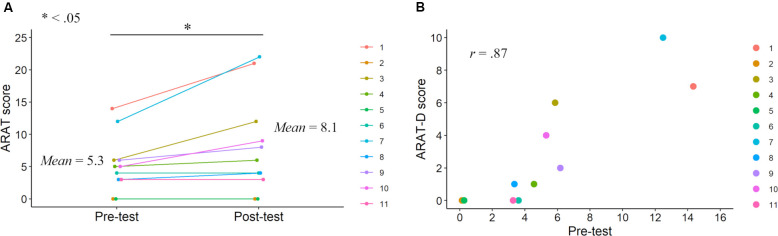
ARAT scores in the pre- and post-tests. ARAT score improvements after the KINVIS therapy with TherEx reported by Kaneko et al. (2019) and their individual differences **(A**). The relationship between the ARAT-D (degree of the improvements from the pre- to post-tests) and pre-test scores **(B)**.

Before the main analyses, we explored the characteristics of upper limb motor functions (FMA and ARAT scores). These investigations contributed to constituting the mediation model, wherein paths were provided to variables representing motor function improvements after KINVIS therapy with TherEx. Using paired t-tests, significant differences were previously reported in the FMA and ARAT total scores between the pre- and post-tests (Kaneko et al., 2019; Figures 2, 3). Based on these results, we conducted correlation analyses to investigate the relationship between the pre-test motor function scores and score improvements from the pre-test to the post-test (FMA-D or ARAT-D). Regarding the ARAT-F or ARAT-S scores, we conducted the same analyses as above (i.e., paired t-tests and correlation analyses). The analyses were conducted using R software (version 4.0.2).

#### Path Analysis Comprising the Mediation Model

After the above analyses, we conducted one of the main investigations, investigating whether improvements in upper limb motor functions after KINVIS therapy with TherEx were induced through spasticity improved by therapy. A path analysis (mediation model) in structural equation modeling was conducted with KINVIS therapy (dummy variable: 0 or 1) as an independent variable; MAS as a mediation variable; FMA, ARAT-F, and ARAT-S scores as dependent variables. This analysis utilized the pre- and post-tests scores. Based on the t-tests and correlation analysis results, we drew paths from the KINVIS to the MAS, FMA, and ARAT-F and from the MAS to the FMA, ARAT-F, and ARAT-S. We evaluated whether this model fit the data by a chi-square test and other model fit indices, such as the root mean square error of approximation (RMSEA), comparative fit index (CFI), or Tucker-Lewis index (TLI). If these indices showed a good fit, we accepted the model and proceeded to interpret the path coefficients. Path coefficients were estimated by the robust weighted least squares, suitable for analyzing the model including an ordinal variable (Muthén, 1984). These analyses were conducted using M-plus (version 8.5).

#### Path Analyses Comprising the Multiple Regression Models

Next, we investigated the relationship between the three rs-FC (i.e., z-scores of the aIPS-uIPS, aSMG-Vermis, and uIPL-uPMd) and upper limb motor functions. We analyzed the pre-test scores of their variables to determine the characteristics of patients with severe post-stroke impairments. Significant correlations between their rs-FC and motor functions (i.e., FMA or ARAT) have been previously reported (Kaneko et al., 2019). To control the rs-FC influences other than the one of interest among them, we conducted a path analysis (multiple regression model) with z-scores of the pre-test aIPS-uIPS, aSMG-Vermis, and uIPL-uPMd as independent variables and the pre-test FMA, ARAT-F, and ARAT-S scores as dependent variables. We drew paths from three independent variables (i.e., three rs-FC) for all three dependent variables.

Finally, we investigated whether pre-test rs-FC scores were associated with the degree of motor function improvements after intervention (i.e., explored the improvement potential in terms of rs-FC). We analyzed pre-test rs-FC scores as independent variables and the difference in motor function scores between pre- and post-tests as dependent variables. In this analysis, variables with significant differences in motor function scores between the pre- and post-tests were adopted as dependent variables (i.e., FMA-D and ARAT-FD, see “Results” section). We drew paths from three independent variables (three rs-FC) for all dependent variables.

These models were saturated, which completely fit the data. Path coefficients were estimated by the robust maximum likelihood, which allowed an accurate estimation regardless of multivariate normal distribution (Yuan and Bentler, 2000). These analyses were conducted using M-plus (version 8.5).

## Results

### Upper Limb Motor Function Characteristics

Correlation analysis revealed no significant correlation between FMA-D and pre-test FMA (*r* = 0.37, *p* = 0.27; Figure 2), whereas a significant positive correlation was observed between the ARAT-D and pre-test ARAT scores (*r* = 0.87, *p* < 0.001; Figure 3). This result indicates that the higher the pre-test scores, the greater the improvements after the interventions.

**Figure 4:**
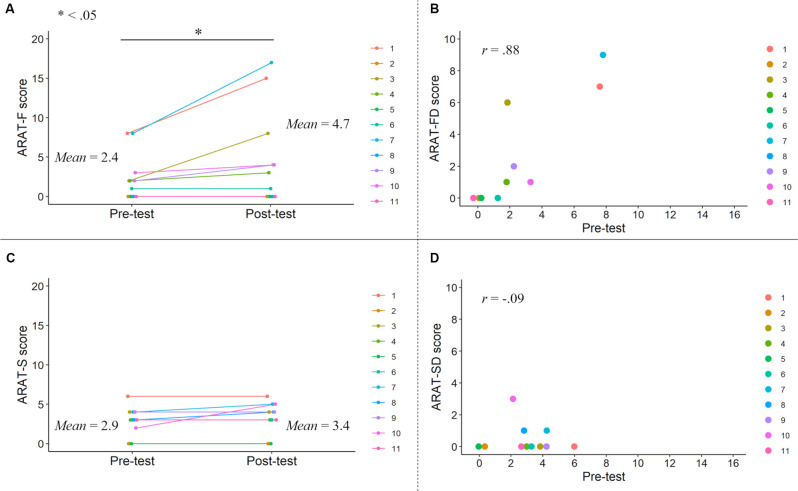
ARAT-F and ARAT-S scores in the pre- and post-tests. ARAT-F score improvements after the KINVIS therapy with TherEx and their individual differences **(A)**. The relationship between the ARAT-FD (degree of the improvements from the pre- to post-tests) and ARAT-F pre-test scores **(B)**. The changes of the ARAT-S scores after the KINVIS therapy with TherEx in individuals **(C)**. The relationship between the ARAT-SD (degree of the improvements from the pre- to post-tests) and ARAT-S pre-test scores **(D)**. The ARAT-F score referred to the total score of the grasp, grip, and pinch subscales. The ARAT-S score referred to the score of the gross movement subscale.

A paired t-test revealed a significant difference between the pre- and post-test ARAT-F scores (*t* = 2.36, *df* = 10, *p* = 0.040, Hedges’g (Hedges, 1981) = 0.52; Figure 4). Correlation analysis revealed a significant positive correlation between the ARAT-FD and pre-test ARAT-F scores (*r* = 0.88, *p* < 0.001), consistent with those of the total ARAT scores. A paired t-test revealed similar pre- and post-test ARAT-S scores (*t* = 1.61, *df* = 10, *p* = 0.14, *g* = 0.26; Figure 4). Correlation analysis revealed similar ARAT-SD and pre-test ARAT-S scores (*r* = −0.094, *p* = 0.78). These results are summarized in Supplementary Table 1.

### Effects of KINVIS Therapy With TherEx Mediated by Spasticity

**Figure 5:**
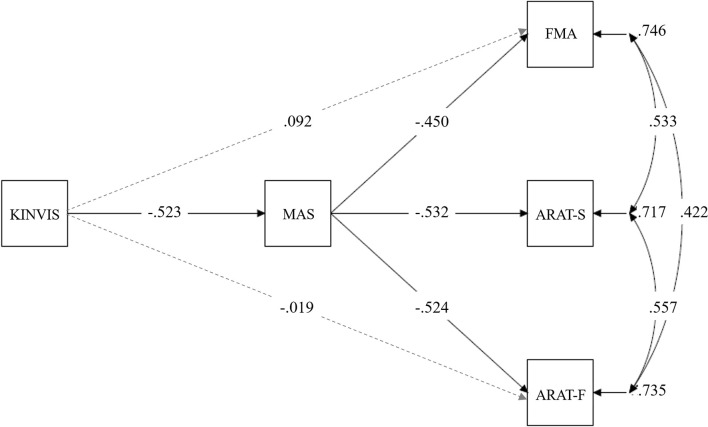
Mediation model in the KINVIS, MAS, FMA, ARAT-F, and ARAT-S. The KINVIS referred to the kinesthetic illusion therapy with TherEx, which was transformed to the dummy variable in this model: before (0) and after (1) the interventions. The ARAT-F referred to the total score of the grasp, grip, and pinch subscales. The ARAT-S referred to the score of the gross movement subscale. Solid lines show paths with significant standardized coefficients, and broken lines show paths with nonsignificant standardized coefficients.

Based on the above results, in structural equation modeling, we drew paths from the KINVIS to the MAS, FMA, and ARAT-F, but not to the ARAT-S (Figure 5); no significant improvement in the ARAT-S score after KINVIS therapy with TherEx was observed (Figure 4). To investigate spasticity influences, we drew paths from the MAS to the FMA, ARAT-F, and ARAT-S. This model fit the data very well; a chi-square test of model fit was not significant (χ^2^ = 1.03, *df* = 1, *p* = 0.31; other model fit indices: RMSEA = 0.037, CFI = 0.99, TLI = 0.98). A path analysis revealed significant standardized path coefficients (*β*) from the KINVIS to the MAS (*β* = −0.52, *p* = 0.003) from the MAS to the FMA, *β* = −0.45, *p* = 0.013, ARAT-S, *β* = −0.53, *p* < 0.001, and ARAT-F, *β* = −0.52, *p* = 0.014, and non-significant standardized coefficients from the KINVIS to the FMA, *β* = 0.092, *p* = 0.72, and ARAT-F, *β* = −0.019, *p* = 0.95. Regarding the indirect KINVIS effects on the FMA and ARAT-F (product of KINVIS to the MAS and MAS to FMA/ARAT-F path coefficients), a bootstrap method (resampling = 2000) revealed bias-corrected 95% confidence intervals in the FMA (0.045, 0.53) and ARAT-F (0.029, 0.61). This model indicated that the KINVIS decreased the MAS score, indirectly improving the FMA and ARAT scores. Supplementary Table 2 presents details of these results.

### Relationship Between the rs-FC and Motor Function Before Intervention

**Figure 6:**
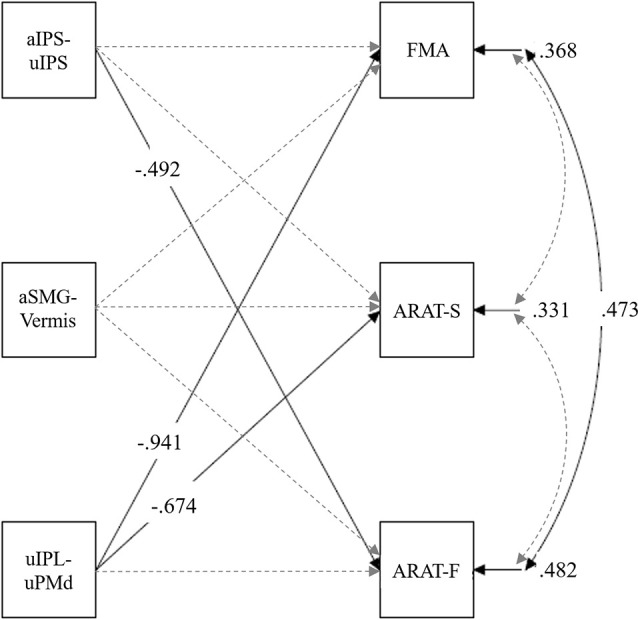
Multiple regression model in pre-test rs-FC and upper limb motor functions. The ARAT-F referred to the total score of the grasp, grip, and pinch subscales. The ARAT-S referred to the score of the gross movement subscale. Solid lines show paths with significant standardized coefficients, and broken lines show nonsignificant paths. Only significant coefficients were shown for clarity (Supplementary Table 3 shows all path coefficients). aIPS-uIPL: rs-FC between the inferior parietal sulcus in the affected and unaffected hemispheres. aSMG-Vermis: rs-FC between the supramarginal gyrus in the affected hemisphere and the cerebellar vermis. uIPL-uPMd: rs-FC between the inferior parietal lobule in the unaffected hemisphere and dorsal premotor cortex in the unaffected hemisphere.

Before a path analysis (multiple regression model), the variance inflation factors of the independent variables within the standard range (2.12, 1.02, and 2.11 in aIPS-uIPS, aSMG-Vermis, and uIPL-uPMd, respectively) were confirmed. A path analysis was conducted with z-scores of the pre-test aIPS-uIPS, aSMG-Vermis, and uIPL-uPMd as independent variables, and the pre-test FMA, ARAT-F, and ARAT-S scores as dependent variables. This analysis revealed significant standardized path coefficients (*β*) from the aIPS-uIPS to the ARAT-F, *β* = −0.49, *p* = 0.024, and from the uIPL-uPMd to the FMA, *β* = −0.94, *p* < 0.001, and ARAT-S, *β* = −0.67, *p* = 0.006 (Figure 6). Supplementary Table 3 lists the details of these results.

### Relationship Between the rs-FC and Motor Function Improvements

**Figure 7:**
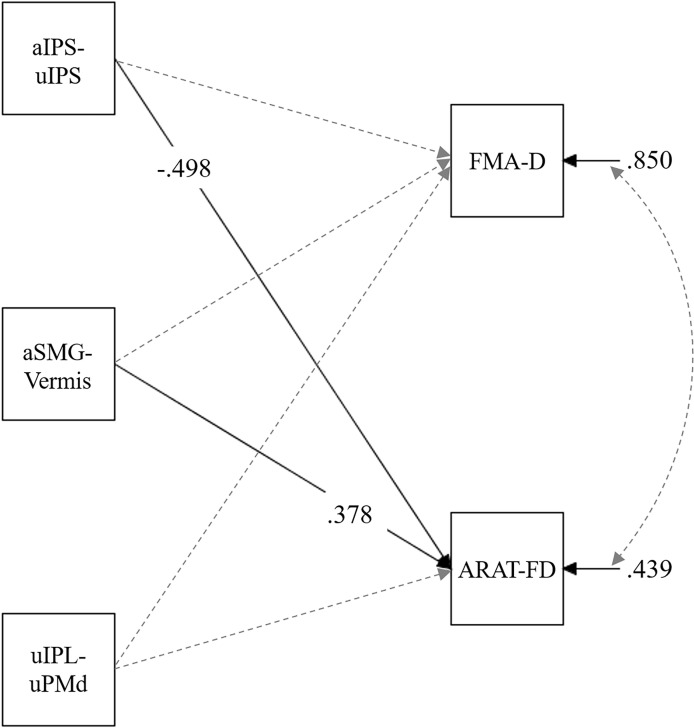
Multiple regression model in pre-test rs-FC and score improvements of upper limb motor function between the pre- and post-test. The FMA-D referred to the degree of improvement between the pre- and post-test in the FMA score. The ARAT-FD referred to the degree of improvement between the pre- and post-test regarding the total score for grasp, grip, and pinch subscales. Solid lines show paths with significant standardized coefficients, and broken lines show nonsignificant paths. Only significant coefficients are shown for clarity (Supplementary Table 4 shows all path coefficients). aIPS-uIPL: rs-FC between the inferior parietal sulcus in the affected and unaffected hemispheres. aSMG-Vermis: rs-FC between the supramarginal gyrus in the affected hemisphere and the cerebellar vermis. uIPL-uPMd: rs-FC between the inferior parietal lobule in the unaffected hemisphere and dorsal premotor cortex in the unaffected hemisphere.

Path analysis was conducted using z-scores of the pre-test aIPS-uIPS, aSMG-Vermis, and uIPL-uPMd as independent variables, and the FMA-D and ARAT-FD scores (i.e., score improvements between the pre-test to the post-test) as dependent variables; the ARAT-SD score was not used in this analysis because no significant difference in score was observed between the pre- and post-tests (Figure 4). This analysis revealed significant standardized path coefficients (*β*) from the aIPS-uIPS to the ARAT-FD, *β* = −0.50, *p* = 0.014, and from the aSMG-Vermis to the ARAT-FD, *β* = 0.38, *p* = 0.019 (Figure 7). Supplementary Table 4 lists the details of these results.

## Discussion

We aimed to investigate the complex relationships among KINVIS therapy with TherEx, spasticity, upper limb motor functions, and rs-FC (i.e., aIPS-uIPS, aSMG-Vermis, or uIPL-uPMd) observed previously (Kaneko et al., 2019). To achieve this, we conducted two main investigations by performing path analyses in structural equation modeling with data from the previous clinical trial (Kaneko et al., 2019). First, we investigated whether the effects of KINVIS therapy with TherEx on motor function improvements were induced through improved spasticity. Second, the relationships among the three rs-FC and motor functions before the interventions were analyzed without spurious correlations between their variables. Consequently, we provide three main findings with a hypothesis for future, large-scale, clinical trials.

### Effects of KINVIS Therapy With TherEx Mediated by Spasticity

In the present study, we demonstrated that KINVIS therapy with TherEx decreased the MAS score that improved the FMA, ARAT-F, and ARAT-S scores (indirect effect of the KINVIS therapy with TherEx). Interestingly, in this mediation model, no significant direct effects from KINVIS to FMA and ARAT-F were observed, although the paired t-tests revealed significant improvements in the FMA and ARAT scores. These results, therefore, suggest that the effects of KINVIS therapy with TherEx on upper limb motor functions were mediated by spasticity. Considering that the patients underwent KINVIS therapy with TherEx for just 10 days, these interventions induced their potential spasticity-masked motor functions. In severe cases, motor function recovery can be smaller than in moderate or mild ones (Vliet et al., 2020). Besides, spasticity can disturb therapeutic exercises for severe cases, e.g., by obscuring motor learning (Subramanian et al., 2018). If the KINVIS therapy improved spasticity, its therapy likely facilitated the effects of TherEx post KINVIS. These combined effects may explain the improvements in motor function in severe cases observed in the previous clinical trial (Kaneko et al., 2019).

How did the KINVIS therapy with TherEx enable improvement of spasticity? To dissect this mechanism, evidence regarding mirror therapy, to treat paretic upper limb in post-stroke patients (Pérez-Cruzado et al., 2017), may help. Mirror therapy causes kinesthetic illusions such as KINVIS; however, few studies have reported significant improvements in spasticity after mirror therapy (Yavuzer et al., 2008; Samuelkamaleshkumar et al., 2014; Pérez-Cruzado et al., 2017). This difference in the effect on spasticity may be due to differences in neurological reactions or maneuvers between the KINVIS and mirror therapies. A systematic review on neuroimaging reported brain regions activated by mirror therapy, such as the superior parietal and fusiform regions (Deconinck et al., 2015). These two regions were consistent with those activated in experiencing KINVIS (Kaneko et al., 2015) and therefore possibly associated with kinesthetic illusions. The KINVIS experiences can activate premotor regions including the supplementary motor area and basal ganglia (Kaneko et al., 2015), not seen in mirror therapy, with little evidence that its therapy activates the mirror neuron system (Deconinck et al., 2015), although KINVIS can be interpreted to be driven by the contralateral hemispherical mirror neuron system. These neurological differences in inducing kinesthetic illusions may be related to the different effects of KINVIS and mirror therapies on spasticity.

The activation of the premotor area, supplementary motor area, and/or network between the association motor and parietal areas may contribute to the effects of KINVIS therapy on spasticity. However, since the previous clinical study (Kaneko et al., 2019) did not measure brain activity during KINVIS, this possibility should be investigated further. KINVIS requires no voluntary movements, even in a non-paralyzed upper limb. In mirror illusion, however, proprioceptive input from non-paralyzed hand movements can affect the kinesthetic illusion (Chancel et al., 2016). Although the non-paralyzed hand movements may reinforce abnormal interhemispheric inhibition (Murase et al., 2004; Nowak et al., 2009), this issue is excluded in KINVIS. Whether these differences explain the mechanism of the effect on spasticity remains unclear; however, these may characterize the clinical effects of KINVIS. This clinical effect is noteworthy and highlights the necessity to explore its mechanism. Recent animal studies highlight the importance of ipsilateral premotor/supplementary-cortico-reticulospinal tract hyperexcitability from the unaffected motor cortex as a result of disinhibition after stroke (Li et al., 2019). Li et al. (2019) suggested a theoretical account that disinhibition after stroke in the premotor area and the supplementary area may cause spasticity and related movement impairments. The enhanced excitability of motor associated areas in the affected hemisphere and lower activity in the unaffected side are possibly remarkable points in managing spasticity. Further study to approach this pathophysiological account may be needed.

Our results also exhibited significant correlations among the FMA and ARAT residual variances, indicating other factors related to variances in the FMA and ARAT scores unexplained by the variables included in this model. This is unsurprising because upper limb motor functions are affected not only by spasticity but also by many factors, such as lesion sites. Regarding the changes in their scores, the present study suggests the importance of considering the pre-intervention motor function scores (at least the ARAT), since a significant correlation between the degree of improvement and pre-test ARAT score was observed. Although the results revealed no significant correlation in the FMA, such correlation could emerge by including patients with moderate or mild motor deficits (Figure 2). Brain activities, including rs-FC, may be associated with KINVIS therapy effects (Kaneko et al., 2015), although our patient sample size was too small to analyze a complex model including their variables. The indirect effect observed in our study, however, can contribute to future model construction, allowing us to explore the KINVIS effect mechanisms.

### Relationship Between the rs-FC and Motor Functions Before the Intervention

We demonstrated significant coefficients from the aIPS-uIPS to the ARAT-F and from the uIPL-uPMd to the FMA and ARAT-S. The former indicates that the rs-FC of aIPS-uIPS may be associated with skilled motor functions of the hands and fingers. Some studies reported that the motor imagery of hand or finger movements activated the parietal areas (Parsons et al., 1995; Stephan et al., 1995). Other evidence indicated that their activities during the motor imagery were similar to those just before performing finger movements, suggesting that their activities are involved in motor preparations (Hanakawa et al., 2003, 2008). According to these findings, the functional connectivity between aIPS and uIPS might play an important role in performing hand and finger movements. In fact, patients with damage in these regions can have difficulties in performing hand and finger movements and recognizing self-body and movements, even though the lesions do not extend to the corticospinal tract (apraxia; Appenzeller and Hanson, 1966). However, the present study illustrated that the stronger the rs-FC of aIPS-uIPS, the weaker the finger motor functions (i.e., negative correlation). This might be due to the strong rs-FC between parietal regions related to motor performances (when motor imageries and preparations are unnecessary) and/or that parietal regions should interact with ipsilateral occipital or frontal regions, but the connectivity in our study did not do so (connectivity between contralateral IPSs). If so, our results suggest a deviation of this functional connectivity from a normal one. This deviation could disturb the hand and finger motor performances.

The other significant coefficients (i.e., from the uIPL-uPMd to the FMA and ARAT-S) indicate that the rs-FC of uIPL-uPMd may be associated with the gross motor functions of the shoulders and elbows. In their gross movements, such as reaching, the motor intentions can arise from the projection through the superior longitudinal fasciculus II and III from the inferior parietal to premotor cortices (Graziano et al., 1994; Rizzolatti et al., 1998). Moreover, gross movements require proximal or trunk muscles to be controlled. Some studies have suggested that the premotor region has bilateral projections to the reticular formation (i.e., cortico-reticular pathway; Matsuyama and Drew, 1997; Kably and Drew, 1998; Yeo et al., 2012). These pathways might bilaterally project to proximal or trunk muscles and may be involved in the posture control required prior to limb movements (Matsuyama and Drew, 1997; Kably and Drew, 1998). It should be noted that the premotor cortex may also have an ipsilateral projection to proximal or trunk muscles. Our study revealed negative correlations between rs-FC of uIPL-uPMd (i.e., uninvolved hemisphere) and motor functions of the paretic upper limb. Given that patients often compensate for motor functions on paretic limbs by the brain functions in the uninvolved hemisphere post stroke, these correlations might reflect compensatory brain activities due to severe motor deficits. Considering that the motor impairments of the patients were within a chronic phase, the rs-FC, aIPS-uIPS or uIPL-uPMd, may represent one of the post-stroke adaptations in the brain. To further investigate this possibility, a longitudinal study would be required.

### Relationship Between the rs-FC and Motor Function Improvements

In terms of rs-FC, we explored the baseline characteristics of patients with the potential to improve their motor functions. Regarding the relationship between the pre-test rs-FC and motor function improvements, significant negative coefficients from the aIPS-uIPS to the ARAT-FD and positive coefficients from the aSMG-Vermis to the ARAT-FD were observed. The former indicates that the weaker rs-FC of aIPS-uIPS before the intervention, the greater the finger motor function improvements, suggesting that the patients with weak rs-FC of aIPS-uIPS have the potential to improve their motor functions. This result is consistent with the pre-test data analysis (see Figure 6), suggesting that the strength of this rs-FC may indicate a deviation from a normal one.

For the latter (positive coefficients from the aSMG-Vermis to the ARAT-FD), it should be noted that rs-FC of the aSMG-Vermis was negative (i.e., the aSMG was negatively correlated with the vermis) although rs-FC of aIPS-uIPS and uIPL-uPMd were positive (Kaneko et al., 2019). Therefore, the positive coefficients from the aSMG-Vermis to the ARAT-FD indicate that the weaker negative rs-FC of aSMG-Vermis before the interventions, the greater the finger motor function improvements. The vermis is known to receive somatosensory inputs from the spinal cord and is related to controlling the anti-gravity muscles of the trunk and legs (Lisberger and Thach, 2013). On the other hand, parietal regions including the SMG can be associated with finger motor functions through motor awareness (Desmurget et al., 2009) or multisensory integration (Quinn et al., 2014). Considering the negative functional connectivity between these regions, the activity of the vermis in a resting state could inhibit that of the SMG, thereby disturbing voluntary finger movements. If so, it is likely that the decline of this negative functional connectivity can contribute to improvements of finger motor functions, as shown by the positive coefficients from the aSMG-Vermis to the ARAT-FD. This may also be a topic of interest for future investigation.

### Limitations

Since we utilized the sample data of a previous clinical trial (Kaneko et al., 2019), our study had similar limitations to that of the previous trial. For example, the previous study had no control group and therefore could not determine which of the two treatments (the KINVIS therapy and TherEx) was effective in reducing spasticity (also see Discussion in Kaneko et al., 2019). Moreover, since KINVIS therapy is composed of KINVIS and NMES, there is a possibility that the reduction of spasticity observed in the previous clinical trial (Kaneko et al., 2019) resulted from NMES itself. However, a meta-analysis on the effects of NMES indicated that NMES combined with other interventions can provide a significant effect on spasticity but not NMES alone, suggesting that combining NMES with other interventions can be a treatment option that provides improvements in spasticity (Stein et al., 2015). Moreover, this study showed that the use of NMES for 30 min five times per week for 3–4 weeks was effective, whereas KINVIS therapy was undertaken for 20 min per day for 10 days. Considering these findings, it seems that the reduction of spasticity observed in the previous clinical trial (Kaneko et al., 2019) resulted in NMES and KINVIS with TherEx. The improvement of motor functions through reducing spasticity may have been caused by the effect of this combination. This possibility should be further investigated by future clinical trials with a control group.

Conversely, the previous sample allowed us to benefit from its clinical characteristics; this sample consisted of patients with severe motor deficits in the chronic phase. For example, our study indicates that in such cases, the fact that the pre-test FMA and ARAT-S scores (gross motor functions of shoulders and elbows) did not predict their improvements, but rather spasticity, might be critical. We acknowledge the importance of using new samples. However, it is also true that conducting a clinical trial is very costly, especially in severe cases. For new studies, the use of previous sample data can be reasonable in some cases (e.g., clinical trials or meta-analysis studies).

Second, the models investigated in this study did not comprehensively include variables due to the small sample size. A complex and comprehensive model including latent variables needs a large sample size for its analysis (Bentler and Chou, 1987). Therefore, we adopted simple models and focused on path coefficients. To identify the relationship among KINVIS therapy, motor functions, and rs-FC, a further study comparing multiple models from the perspective of model fit indices and theory is needed.

## Conclusions

In summary, our study provides three main findings. First, the effects of KINVIS therapy with TherEx on upper limb motor function can be mediated by spasticity. Second, the results suggest that rs-FC of aIPS-uIPS is associated with the skilled motor functions of the hands and fingers, and that rs-FC of uIPL-uPMd is involved in the gross motor functions of the shoulders and elbows. Finally, weaker rs-FC of aIPS-uIPS or aSMG-Vermis might reflect potentials to improve post-stroke impairments in using KINVIS therapy with TherEx. These findings help construct hypothetical models examined in large-scale future clinical trials and underscore the necessity to examine the integrated model in which brain activities account for the indirect effect of KINVIS therapy with TherEx.

## Data Availability Statement

The original contributions presented in the study are included in the article/Supplementary Material, further inquiries can be directed to the corresponding author.

## Ethics Statement

The studies involving human participants were reviewed and approved by Shonan Keiiku Hospital Ethics Committee. The patients/participants provided their written informed consent to participate in this study.

## Author Contributions

FK and ML received funding. FK managed the project and resources, supervision, conceptualization, partial writing—review and editing. YM and MY analyzed clinical and fMRI data. YM and all other authors wrote the article. All authors contributed to the article and approved the submitted version.

## Conflict of Interest

FK and MK are the founding scientists of the startup company INTEP Inc. for the social implementation of university research results. ML is currently an advisor of Connect Inc. These companies do not have any relationship with the device or setup used in the present study. FK received license fees from Inter Reha Co., Ltd. The remaining authors declare that the research was conducted in the absence of any commercial or financial relationships that could be construed as a potential conflict of interest.

## Publisher’s Note

All claims expressed in this article are solely those of the authors and do not necessarily represent those of their affiliated organizations, or those of the publisher, the editors and the reviewers. Any product that may be evaluated in this article, or claim that may be made by its manufacturer, is not guaranteed or endorsed by the publisher.

## Abbreviations

aIPS: Inferior parietal sulcus in the affected hemisphere
ARAT: Action Research Arm Test
ARAT-F: finger-related ARAT score
ARAT-FD: degree of ARAT-F score improvements from the pre-test to the post-test
ARAT-S: shoulder-related ARAT score
ARAT-SD: degree of ARAT-S score improvements from the pre-test to the post-test
aSMG: supramarginal gyrus in the affected hemisphere
FMA: Fugl-Meyer Assessment
FMA-D: degree of FMA score improvements from the pre-test to the post-test
KINVIS: kinesthetic illusion of segmental body part induced by visual stimulation
MAS: modified Ashworth Scale
rs-FC: resting-state functional connectivity
TherEx: therapeutic exercise
uIPL: inferior parietal lobule in the unaffected hemisphere
uIPS: inferior parietal sulcus in the unaffected hemisphere
uPMd: dorsal premotor cortex in the unaffected hemisphere.

## References

[B1] Altschuler E. L. Wisdom S. B. Stone L. Foster C. Galasko D. Llewellyn D. M.. (1999). Rehabilitation of hemiparesis after stroke with a mirror. Lancet 353, 2035–2036. 10.1016/s0140-6736(99)00920-4 10376620

[B2] Aoyama T. Kanazawa A. Kohno Y. Watanabe S. Tomita K. Kimura T.. (2020). Feasibility case study for treating a patient with sensory ataxia following a stroke with kinesthetic illusion induced by visual stimulation. Prog. Rehabil. Med. 5:20200025. 10.2490/prm.20200025 33134593 PMC7591318

[B3] Aoyama T. Kaneko F. Hayami T. Shibata E. (2012). The effects of kinesthetic illusory sensation induced by a visual stimulus on the corticomotor excitability of the leg muscles. Neurosci. Lett. 514, 106–109. 10.1016/j.neulet.2012.02.069 22402187

[B4] Appenzeller O. Hanson J. C. (1966). Parietal ataxia. Arch. Neurol. 15, 264–269. 10.1001/archneur.1966.00470150042007 5912006

[B5] Barros Galvão S. C. Borba Costa dos Santos R. Borba dos Santos P. Cabral M. E. Monte-Silva K. (2014). Efficacy of coupling repetitive transcranial magnetic stimulation and physical therapy to reduce upper-limb spasticity in patients with stroke: a randomized controlled trial. Arch. Phys. Med. Rehabil. 95, 222–229. 10.1016/j.apmr.2013.10.023 24239881

[B6] Bentler P. M. Chou C. P. (1987). Practical issues in structural modeling. Sociol. Methods Res. 16, 78–117. 10.1177/0049124187016001004

[B7] Chancel M. Brun C. Kavounoudias A. Guerraz M. (2016). The kinaesthetic mirror illusion: how much does the mirror matter?. Exp. Brain Res. 234, 1459–1468. 10.1007/s00221-015-4549-5 26790422

[B8] Deconinck F. J. A. Smorenburg A. R. P. Benham A. Ledebt A. Feltham M. G. Savelsbergh G. J. P. (2015). Reflections on mirror therapy: a systematic review of the effect of mirror visual feedback on the brain. Neurorehabil. Neural Repair. 29, 349–361. 10.1177/1545968314546134 25160567

[B9] Desmurget M. Reilly K. T. Richard N. Szathmari A. Mottolese C. Sirigu A. (2009). Movement intention after parietal cortex stimulation in humans. Science 324, 811–813. 10.1126/science.1169896 19423830

[B10] Dohle C. Püllen J. Nakaten A. Küst J. Rietz C. Karbe H. (2009). Mirror therapy promotes recovery from severe hemiparesis: a randomized controlled trial. Neurorehabil. Neural Repair 23, 209–217. 10.1177/1545968308324786 19074686

[B11] Graziano M. S. Yap G. S. Gross C. G. (1994). Coding of visual space by premotor neurons. Science 266, 1054–1057. 10.1126/science.7973661 7973661

[B12] Hanakawa T. Dimyan M. A. Hallett M. (2008). Motor planning, imagery and execution in the distributed motor network: a time-course study with functional MRI. Cereb. Cortex. 18, 2775–2788. 10.1093/cercor/bhn036 18359777 PMC2583155

[B13] Hanakawa T. Immisch I. Toma K. Dimyan M. A. Van Gelderen P. Hallett M. (2003). Functional properties of brain areas associated with motor execution and imagery. J. Neurophysiol. 89, 989–1002. 10.1152/jn.00132.2002 12574475

[B14] Hedges L. V. (1981). Distribution theory for Glass’s estimator of effect size and related estimators. J. Educ. Stat. 6, 107–128. 10.3102/10769986006002107

[B15] Kably B. Drew T. (1998). Corticoreticular pathways in the cat. I. Projection patterns and collaterization. J. Neurophysiol. 80, 389–405. 10.1152/jn.1998.80.1.389 9658059

[B16] Kaneko F. Blanchard C. Lebar N. Nazarian B. Kavounoudias A. Romaiguère P. (2015). Brain regions associated to a kinesthetic illusion evoked by watching a video of one’s own moving hand. PLoS One 10:e0131970. 10.1371/journal.pone.0131970 26287488 PMC4544853

[B17] Kaneko F. Hayami T. Aoyama T. Kizuka T. (2014). Motor imagery and electrical stimulation reproduce corticospinal excitability at levels similar to voluntary muscle contraction. J. Neuroeng. Rehabil. 11:94. 10.1186/1743-0003-11-94 24902891 PMC4113028

[B18] Kaneko F. Inada T. Matsuda N. Shibata E. Koyama S. (2016a). Acute effect of visually induced kinesthetic illusion in patients with stroke: a preliminary report. Int. J. Neurorehabil. 3, 1–6. 10.4172/2376-0281.1000212

[B20] Kaneko F. Shibata E. Hayami T. Nagahata K. Aoyama T. (2016b). The association of motor imagery and kinesthetic illusion prolongs the effect of transcranial direct current stimulation on corticospinal tract excitability. J. Neuroeng. Rehabil. 13:36. 10.1186/s12984-016-0143-8 27079199 PMC4832525

[B19] Kaneko F. Murakami T. Onari K. Kurumadani H. Kawaguchi K. (2003). Decreased cortical excitability during motor imagery after disuse of an upper limb in humans. Clin. Neurophysiol. 114, 2397–2403. 10.1016/s1388-2457(03)00245-1 14652100

[B21] Kaneko F. Shindo K. Yoneta M. Okawada M. Akaboshi K. Liu M. (2019). A case series clinical trial of a novel approach using augmented reality that inspires self-body cognition in patients with stroke: effects on motor function and resting-state brain functional connectivity. Front. Sys. Neurosci. 13:76. 10.3389/fnsys.2019.00076 31920571 PMC6929676

[B22] Kaneko F. Yasojima T. Kizuka T. (2007). Kinesthetic illusory feeling induced by a finger movement movie effects on corticomotor excitability. Neuroscience 149, 976–984. 10.1016/j.neuroscience.2007.07.028 17935897

[B23] Klamroth-Marganska V. Blanco J. Campen K. Curt A. Dietz V. Ettlin T.. (2014). Three-dimensional, task-specific robot therapy of the arm after stroke: a multicentre, parallel-group randomised trial. Lancet Neurol. 13, 159–166. 10.1016/S1474-4422(13)70305-3 24382580

[B24] Li S. Chen Y. T. Francisco G. E. Zhou P. Rymer W. Z. (2019). A unifying pathophysiological account for post-stroke spasticity and disordered motor control. Front. Neurol. 10:468. 10.3389/fneur.2019.00468 31133971 PMC6524557

[B25] Lisberger S. G. Thach W. T. (2013). “The cerebellum,” in Principles of Neural Science, eds Kandel E. R. Koester J. D. Mack S. H. Siegelbaum S. A. (New York: McGraw-Hill), 960–981.

[B26] Matsuyama K. Drew T. (1997). Organization of the projections from the pericruciate cortex to the pontomedullary brainstem of the cat: a study using the anterograde tracer Phaseolus vulgaris-leucoagglutinin. J. Comp. Neurol. 389, 617–641. 10.1002/(sici)1096-9861(19971229)389:4<617::aid-cne6>3.0.co;2-3 9421143

[B27] Murase N. Duque J. Mazzocchio R. Cohen L. G. (2004). Influence of interhemispheric interactions on motor function in chronic stroke. Ann. Neurol. 55, 400–409. 10.1002/ana.10848 14991818

[B28] Muthén B. (1984). A general structural equation model with dichotomous, ordered categorical and continuous latent variable indicators. Psychometrika 49, 115–132. 10.1007/BF02294210

[B29] Nowak D. A. Grefkes C. Ameli M. Fink G. R. (2009). Interhemispheric competition after stroke: brain stimulation to enhance recovery of function of the affected hand. Neurorehabil. Neural Repair 23, 641–656. 10.1177/1545968309336661 19531606

[B30] Okawada M. Kaneko F. Shindo K. Yoneta M. Sakai K. Okuyama K.. (2020). Kinesthetic illusion induced by visual stimulation influences sensorimotor event-related desynchronization in stroke patients with severe upper-limb paralysis: a pilot study. Restor. Neurol. Neurosci. 38, 455–465. 10.3233/RNN-201030 33325415

[B31] Page S. J. Levine P. Leonard A. (2007). Mental practice in chronic stroke. Stroke 38, 1293–1297. 10.1161/01.STR.0000260205.67348.2b 17332444

[B32] Parsons L. M. Fox P. T. Downs J. H. Glass T. Hirsch T. B. Martin C. C.. (1995). Use of implicit motor imagery for visual shape discrimination as revealed by PET. Nature 375, 54–58. 10.1038/375054a0 7723842

[B33] Pérez-Cruzado D. Merchán-Baeza J. A. González-Sánchez M. Cuesta-Vargas A. I. (2017). Systematic review of mirror therapy compared with conventional rehabilitation in upper extremity function in stroke survivors. Aust. Occup. Ther. J. 64, 91–112. 10.1111/1440-1630.12342 28032336

[B34] Pollock A. Farmer S. E. Brady M. C. Langhorne P. Mead G. E. Mehrholz J.. (2014). Interventions for improving upper limb function after stroke. Cochrane Database Syst. Rev. 2014:CD010820. 10.1002/14651858.CD010820.pub2 25387001 PMC6469541

[B35] Pundik S. McCabe J. Skelly M. Tatsuoka C. Daly J. J. (2019). Association of spasticity and motor dysfunction in chronic stroke. Ann. Phys. Rehabil. Med. 62, 397–402. 10.1016/j.rehab.2018.07.006 30099149

[B36] Quinn B. T. Carlson C. Doyle W. Cash S. S. Devinsky O. Spence C.. (2014). Intracranial cortical responses during visual-tactile integration in humans. J. Neurosci. 34, 171–181. 10.1523/JNEUROSCI.0532-13.2014 24381279 PMC3866483

[B37] Rizzolatti G. Luppino G. Matelli M. (1998). The organization of the cortical motor system: new concepts. Electroencephalogr. Clin. Neurophysiol. 106, 283–296. 10.1016/s0013-4694(98)00022-4 9741757

[B38] Samuelkamaleshkumar S. Reethajanetsureka S. Pauljebaraj P. Benshamir B. Padankatti S. M. David J. A. (2014). Mirror therapy enhances motor performance in the paretic upper limb after stroke: a pilot randomized controlled trial. Arch. Phys. Med. Rehabil. 95, 2000–2005. 10.1016/j.apmr.2014.06.020 25064777

[B39] Shibata E. Kaneko F. (2019). Event-related desynchronization possibly discriminates the kinesthetic illusion induced by visual stimulation from movement observation. Exp. Brain Res. 237, 3233–3240. 10.1007/s00221-019-05665-1 31630226

[B40] Stein C. Fritsch C. G. Robinson C. Sbruzzi G. Plentz R. D. M. (2015). Effects of electrical stimulation in spastic muscles after stroke: systematic review and meta-analysis of randomized controlled trials. Stroke 46, 2197–2205. 10.1161/STROKEAHA.115.009633 26173724

[B41] Stephan K. M. Fink G. R. Passingham R. E. Silbersweig D. Ceballos-Baumann A. O. Frith C. D.. (1995). Functional anatomy of the mental representation of upper extremity movements in healthy subjects. J. Neurophysiol. 73, 373–386. 10.1152/jn.1995.73.1.373 7714579

[B42] Subramanian S. K. Feldman A. G. Levin M. F. (2018). Spasticity may obscure motor learning ability after stroke. J. Neurophysiol. 119, 5–20. 10.1152/jn.00362.2017 28904099 PMC5866466

[B43] Vliet R. van der. Selles R. W. Andrinopoulou E. R. Nijland R. Ribbers G. M. Frens M. A.. (2020). Predicting upper limb motor impairment recovery after stroke: a mixture model. Ann. Neurol. 87, 383–393. 10.1002/ana.25679 31925838 PMC7065018

[B60] Whitfield-Gabrieli S. Nieto-Castanon A. (2012). Conn: a functional connectivity toolbox for correlated and anticorrelated brain networks. Brain Connect. 2, 125–141. 10.1089/brain.2012.0073 22642651

[B44] Wolf S. L. Winstein C. J. Miller J. P. Taub E. Uswatte G. Morris D.. (2006). Effect of constraint-induced movement therapy on upper extremity function 3 to 9 months after stroke: the EXCITE randomized clinical trial. JAMA 296, 2095–2104. 10.1001/jama.296.17.2095 17077374

[B45] Yahagi S. Shimura K. Kasai T. (1996). An increase in cortical excitability with no change in spinal excitability during motor imagery. Percept. Mot. Skills 83, 288–290. 10.2466/pms.1996.83.1.288 8873203

[B46] Yavuzer G. Selles R. Sezer N. Sütbeyaz S. Bussmann J. B. Köseoğlu F.. (2008). Mirror therapy improves hand function in subacute stroke: a randomized controlled trial. Arch. Phys. Med. Rehabil. 89, 393–398. 10.1016/j.apmr.2007.08.162 18295613

[B47] Yeo S. S. Chang M. C. Kwon Y. H. Jung Y. J. Jang S. H. (2012). Corticoreticular pathway in the human brain: diffusion tensor tractography study. Neurosci. Lett. 508, 9–12. 10.1016/j.neulet.2011.11.030 22197953

[B48] Yuan K. H. Bentler P. M. (2000). Three likelihood-based methods for mean and covariance structure analysis with nonnormal missing data. Sociol. Methodol. 30, 165–200. 10.1111/0081-1750.00078

